# Clinicohistological Characteristics of Patients with Oral Lichenoid Mucositis: A Retrospective Study for Dental Hospital Records

**DOI:** 10.3390/jcm12196383

**Published:** 2023-10-06

**Authors:** Abdullah Alsoghier, Nasser AlMadan, Mohammed Alali, Rana Alshagroud

**Affiliations:** 1 Department of Oral Medicine and Diagnostic Sciences, College of Dentistry, King Saud University, Riyadh 12372, Saudi Arabia; ralshagroud@ksu.edu.sa; 2 King Saud University Medical City, King Saud University, Riyadh 12372, Saudi Arabia; nmalmadan@gmail.com (N.A.); dr.matooq@gmail.com (M.A.); 3 Dental Center, Prince Sultan Military Medical City, Riyadh 12233, Saudi Arabia

**Keywords:** mouth diseases, lichen planus, oral, pathology, oral, oral medicine, retrospective studies

## Abstract

Oral lichenoid mucositis (OLM) of the oral mucosa is a histological diagnosis mainly characterised by a band of inflammatory infiltrate in lamina propria and basal cell degeneration. These features describe oral lichen planus or oral lichenoid reaction. However, it could be seen in oral dysplasia. The study aimed to assess the demographics and clinicohistological characteristics of patients with OLM and their relevance to dysplastic changes in the oral mucosa. This was a cross-sectional and retrospective study of archived and electronic records of individuals with histological confirmation of OLM at King Saud University Medical City, Saudi Arabia. The descriptive and correlation assessments were used to describe the demographics and clinicohistological characteristics and their associations, respectively [*p* < 0.05]. The analysis included 140 records of patients with histological confirmation of OLM with a mean age of 47 (±13), and 57% (n = 81) were females. Notably, 40% of patients had at least one medical condition, mainly diabetes mellitus, 74% were asymptomatic, and 52% had lesions in the buccal mucosa. Dysplasia was seen in 18 (12%) of the 140 reviewed records. Regarding the associations between study variables, dysplastic changes were associated with the male gender [*p* = 0.024] and were of no significance with increased age [*p* = 0.594]. Moreover, having oral symptoms was associated with older age [*p* < 0.001], medical history of diabetes [*p* = 0.0132] and hypertension [*p* < 0.001]. The present study findings could help indicate the individuals with histologically confirmed OLM who suffer the most from the clinical disease and have an increased risk of dysplastic changes. Therefore, symptomatic management and long-term follow-up can be planned accordingly.

## 1. Introduction

Oral Lichen planus (OLP) is a chronic autoimmune mucocutaneous disorder affecting the oral cavity and sometimes associated with extraoral involvement of the skin, scalp, nail, and mucous membranes, including oral, genital, oesophageal, ocular, and laryngeal mucosa [1]. It is estimated that the prevalence rate of OLP among the general population is around 0.89%, with variations noticed toward specific age groups and continents [2]. The aetiopathogenesis is likely attributed to cell-mediated hypersensitivity, reactive autoimmune activity triggered by epithelial antigens, microbial agents, and stress [1]. Known triggers include certain medications, dental materials, poor oral health, liver diseases and infections, psychological status, genetic profile and tobacco use [1,3,4,5]. Clinically, OLP could present as asymptomatic lesion/s or with a myriad of symptoms such as oral discomfort or pain, burning sensation, xerostomia, sialorrhea, dysgeusia and sometimes psychosocial comorbidities (e.g., anxiety, depression, and sleep disturbances) [6].

Due to the existing controversies concerning the aetiology and prognosis of OLP and oral lichenoid lesions (OLL), the term ‘oral lichenoid disease’ was used to describe the clinically white linear lines with/without papular, atrophic, erythematous and ulcerative lesion/s affecting the oral mucosa [7].

Oral lichenoid disease, especially with its OLL subtype, is considered an oral premalignant condition with a possible malignant transformation to oral squamous cell carcinoma (OSCC) [8]. There is limited understanding of how malignant transformation (MT) occurs compared to other known oral potentially malignant disorders [9,10]. It is also important that lichenoid mucositis features can be present in other mucosal diseases such as mucus membrane pemphigoid, discoid lupus erythematous, graft-versus-host disease, paraneoplastic pemphigus and chronic ulcerative stomatitis [11,12,13]. Moreover, the histological finding ‘oral lichenoid mucositis’ (OLM) exhibits a band-like feature in the sub-epithelial area, disrupted base membrane and basal cell layer, and possibly dysplastic changes in the form of ‘lichenoid mucositis with dysplasia’ or ‘dysplasia with lichenoid mucositis [9,14,15].

Several international studies assessed the characteristics of patients with OLP/OLL in Brazil [16], Canada [14], China [4], the Czech Republic [17], Spain [18], Thailand [19] and the UK [20]. Salem (1989) has reported the clinical characteristics of 72 patients with OLP and indicated an MT rate of only four individuals over the follow-up period of 3.2 years [21]. This study did not detail the histological characteristics concerning OLP. Furthermore, another KSA-based study focused on hepatitis C virus serology among patients with lichen planus in Jazan [5]. Of note, a recent study that assessed the characteristics of 50 patients with OLP noted higher female prevalence, around one-third of them with diabetes and hypertension, and 64% had no oral symptoms [22]. Nevertheless, the study was limited by not detailing the histological features and relatively low sample size.

Therefore, the present study aimed to retrospectively assess the clinicopathological characteristics of patients who have been histologically diagnosed with OLM at the Dental University Hospital, King Saud University Medical City in Riyadh, Saudi Arabia. This term was used to describe both OLP and OLL due to the challenges in distinguishing both disorders by clinicians and histopathologists [11,13,14].

## 2. Methods

This was a cross-sectional retrospective study of the archived and electronic records of patients histologically diagnosed with either OLM or OLP at the Dental University Hospital, King Saud Medical City (Riyadh, Saudi Arabia) from January 1994 to May 2021. The slides were histologically assessed by trained oral pathologists with relevance to OLM and OLP based on the recognised criteria [1,9]. Records of patients without histopathological confirmation, with oral squamous cell carcinoma and those with multiple missing clinicopathological data were excluded from the analysis [23].

Ethical approval was prospectively obtained from the King Saud University Institutional Review Board (reference: E-22-7134, date: 25 September 2022). All study activities aligned with the Declaration of Helsinki’s ethical principles for medical research involving human subjects and identifiable human material and data [24]. No personal data was collected, and all study investigators were clinicians who received bioethics training certification from the National Committee of BioEthics (NCBE-KACST) at King Abdulaziz City for Science and Technology. The consent for publication was not required by the IRB committee for this retrospective review of hospital records. The collected information included demographic data, clinical characteristics, and histological features. Furthermore, the slides were retrieved from the archive of the histopathology unit and reviewed independently by two pathologists (NA, MA). Disagreements were finally addressed by a senior oral histopathologist (RA). 

### Statistical Analysis and Data Management

Continuous data was presented using mean, standard deviation, and median values. Categorical variables were represented by frequency and percentage. Chi-square and Fisher’s Exact tests were used to assess the statistical significance of cross-tabulations between categorical variables [4]. The Shapiro-Wilk test was used to indicate the normality of the continuous data. The unpaired Student’s *t*-test was employed for continuous variables to compare variables that show normal distribution. Also, the Mann-Whitney U-test was utilised for variables that did not follow a normal distribution [23]. A *p*-value of less than 0.05 was considered statistically significant in all the tests. Data analysis was performed using R [v 4.3.2] and Microsoft Excel [v 16.73].

## 3. Results

Initially, 184 records of patients diagnosed with OLM were considered for analysis. After excluding patient records with multiple missing data and those lacking histological assessments (n = 44), the analysed data included 140 records that were histologically confirmed as OLM included in the analysis. 

The mean and median ages of the 140 patients were 47 (± 13) and 48, aged between 22 and 79. Of these, 81 were females (58%) compared to 59 males (42%). The medical history review indicated that 60% of patients (n = 84) had no reported medical problems at the time of biopsy. The remaining 40% had at least one medical problem, including diabetes millets (n = 27), hypertension (n = 20), dyslipidaemia (n = 11), hypothyroidism (n = 9), cutaneous lichen planus (n = 3), asthma (n = 3), breast cancer (n = 2), cutaneous allergy (n = 2), and depression (n = 2). Also, other medical problems reported included cardiac catheterisation, coronary artery bypass surgery, fatigue, inflammatory bowel disease, liver cirrhosis, peptic ulcer, psoriasis, rheumatoid arthritis, Sjogren’s syndrome, vitamin D deficiency, and xerostomia (n = 1 each). Moreover, 126 out of the 140 patients (90%) were not using tobacco, with the remaining 10% smoking cigarettes (n = 11) combined with smokeless tobacco (n = 1) and previous smokers (n = 2).

Of the 140 reviewed records, the clinical diagnosis was mainly OLP in 96 (68%), followed by OLP/lichenoid reaction (n = 12) and lichenoid reaction (n = 6) in association with leukoplakia (n = 4) or erythroplakia (n = 2). Among these records, the differential diagnosis included mucus membrane pemphigoid (n = 6), pemphigus vulgaris (n = 4), discoid (n = 2) and systemic lupus erythematous (n = 1), and trauma-related keratosis (n = 1). The remaining 26 records (18%) did not include clinical diagnoses with missing data due to old printing/electronic archiving issues (n = 10), or no clinical correlation was mentioned (n = 7). Others were attributed clinically to keratosis (n = 5) or ulceration secondary to trauma (n = 2) and squamous papilloma/multifocal epithelial hyperplasia (n = 2). 

Of note, 74% of patients were asymptomatic compared to the remaining 26% who noted oral burning sensation, pain, pruritus and nausea/vomiting. Regarding the biopsy site, 52% of the patients had it in the buccal mucosa, 11% in the gingiva and 8% in the lateral tongue. Despite the subjectively measured and missing data about consistency, 44% of the lesions were considered by clinicians as soft. Similarly, the documented colour of the lesions was described as ‘white’ in 35% of patients and ‘mixed red and white’ in 25% of patients (Table 1). Furthermore, desquamative gingivitis was reported in only six records, of which 5 were female patients.

Concerning histological assessments, the common findings in the reviewed oral biopsy slides were band-like inflammatory cells (100%), epithelial hyperkeratosis (99%), atrophy (88%), basal cell degeneration (88%), squamatisation (87%), thickening of basement membrane (72%), fibrin deposits (71%), Civatte bodies (60%), melanin incontinence (58%). Regarding the 138 slides with epithelial hyperkeratosis, hyperparakeratosis was found in 80 (57%), hyperorthokeratosis in 17 (12%) and both findings were demonstrated among 41 (29%) (Table 2).

**Figure 1:**
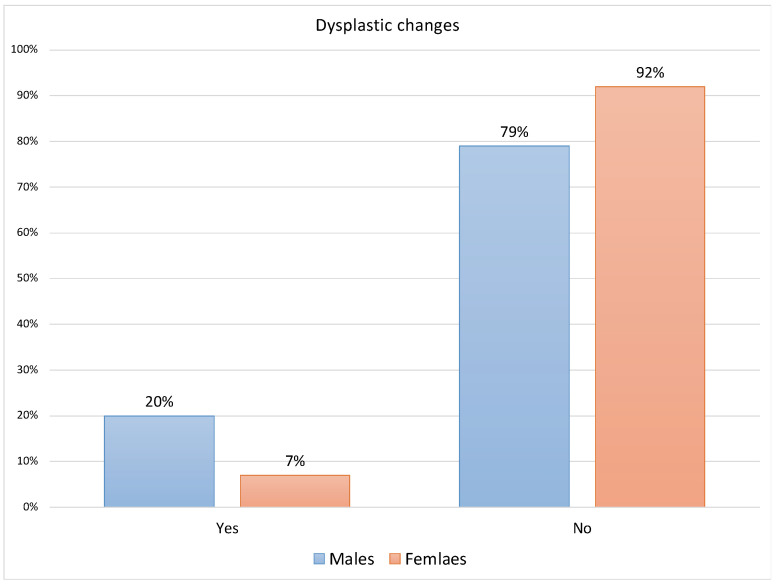
Distribution of dysplastic changes for males and females (n = 140).

Regarding the associations between gender and clinicopathological variables (Supplementary File S1), the Fisher’s Exact test indicated a statistically significant difference between the presence of dysplastic changes/cytological atypia among 12 of the 59 males (20%) compared to 6 of the 81 females (7%) [*p* = 0.024]. Similarly, this gender difference was also significant regarding the lack of dysplasia among 79% and 92% of males and females, respectively [*p* = 0.0473] (Figure 1).

There were no other significant differences observed between gender and each of the sites, symptoms, and histological findings such as squamatisation, basal cell degeneration, grade of inflammation, fibrin deposit, epithelial hyperkeratosis, hypergranulosis, acanthosis, atrophic, Civatte bodies, saw tooth rete ridges, artificial cleft formation, melanin incontinence, thickening of basement membrane and ulceration.

The assessments of associations between medical history and oral symptoms (Table 3) indicated a statistically significant difference between the presence of symptoms and each having a medical history of diabetes mellitus [*p* = 0.0132] and hypertension [*p* < 0.001] compared to those who had no symptoms (33% and 36% compared to 14% and 6%, respectively). Furthermore, 55% and 16% of records with dysplastic changes (n = 18) showed hyperorthokeratosis and hyperparakeratosis compared to 5% and 63% of records without dysplasia (n = 122) [*p* < 0.001], respectively.

Despite the lack of statistical significance by Fisher’s Exact test, 12% of the 124 records with atrophy showed hyperorthokeratosis, while 13% of 15 records without atrophy had hyperorthokeratosis [*p* = 0.999]. Moreover, records with atrophy (58%) were more likely to demonstrate hyperparakeratosis than those without (40%), as indicated by the Chi-square test [*p* = 0.163]. Regarding the associations between the age and study findings, patients with oral symptoms had an increased mean age than those who were asymptomatic at the time of biopsy (53 ± 13 and 45 ± 12, respectively) [*p* < 0.001]. Similarly, patients with ulceration had a higher mean age (54 ± 11) than those without (46 ± 13). The age difference based on the presence of ulcers was statistically significant (*p* = 0.0149) (Table 4).

Although the age difference was not statistically significant, the dysplastic changes were more associated with the older mean age of patients (49 ± 11) compared to patients without dysplasia (47 ± 13) [*p* = 0.594]. There were no significant differences observed in age with regards to squamatisation, basal cell degeneration, fibrin deposit, hypergranulosis, acanthosis, atrophy, Civatte Bodies, saw tooth rete ridges, artificial cleft formation, melanin incontinence and thickening of the basement membrane (Supplementary File S2).

## 4. Discussion

The present study reported key findings related to the demographic, clinical, and histological characteristics of 140 patients with OLM. These can help identify individuals likely to experience oral symptoms and increased risk of dysplastic changes or possibly further progression to OSCC. With the lack of clear definitions and validated histological threshold for diagnosing OLP/OLL, reporting the histological findings could help in clinicopathologic correlation and forming an oral medicine/pathology consensus on diagnosing and managing these conditions [9,23,25].

The mean age of patients presently (47) was relatively younger than that of other OLP patient cohorts in Saudi (48) [22], China and Thailand (50) [4,19], Brazil (54) [16], Czech Republic (55) [17] and Spain (56) [18]. However, the figures from the World Bank mirrored this difference by showing increased percentages of individuals aged 65 and above in these countries compared to that in Saudi Arabia [26]. Notably, the youngest age to receive the among the current study sample (22) was similar to what was found in other patient cohorts in Brazil [16], but higher than other studies reporting ages as young as 10 and 15 [4,23].

OLP in childhood is uncommon and tends to be associated with a familial distribution in 11% of patients aged between 6 and 17 and increased disease activity [27]. Also, the evidence of similar HLA alleles (e.g., HLA-B7) in studies involving familial lichen planus [28] requires further assessments of the genetic makeup and susceptibility in childhood. Clinicians may consider periodic screening for oral mucosal changes that warrant further management despite the lack of evidence of MT among paediatric OLP patients [27].

Whether OLP is more prevalent in patients with diabetes mellitus than the general population remains unclear and contradictory due to different clinical study designs and populations [4,21]. It is not uncommon that systemic disorders with low-grade inflammation and their used medications are associated with changes in the oral mucosa that can mimic or reflect lichenoid changes [29]. OLP management sometimes includes long-term systemic corticosteroids and could increase the risk of diabetes and hypertension in this patient population [30]. Although this association was not demonstrated in retrospective studies [4,21], a systematic review showed a possible increased prevalence of OLP among individuals with diabetes compared to those in the control group [29]. Many of the included studies in this systematic review did not necessarily determine the diabetes subtypes and their diagnostic methods, and some only considered a clinical diagnosis of OLP [29]. Thus, further prospective case-controlled studies are needed to assess these correlations, considering the high prevalence of these diseases in the age groups that often correspond with the OLP onset [2,29].

Although oral soreness was often noted among 47–92% of patients with OLP/OLL [4,16,17,19,20], this was less encountered presently with only 26% of patients indicating soreness. Such findings require careful interpretation as individuals may have received management (e.g., topical or systemic corticosteroid) before referral for histological confirmation of OLP at the present tertiary referral hospital. Furthermore, the present findings were in line with clinical determinants of OLP-related symptoms (e.g., burning sensation, irritation, bleeding and swelling) such as longer disease duration, clinical form (erosive) and increased age [4]. Regarding its severity, males with OLP/OLL were more likely to experience higher severity [23], which was not observed in the present analysis as there were no reported correlations between gender and oral symptoms.

The agreement between the present study findings and other studies, including mainly White/Caucasian patient cohorts [16,20], indicated similar clinicopathological characteristics with an exception toward lower oral soreness reporting rate presently. Pain experience is often perceived differently among different patient ethnicities, considering the multidimensional aspects of pain and their biopsychosocial determinants [31]. Therefore, assessments of cross-cultural differences in disclosing oral soreness and their impact on quality of life are needed to deliver tailored and patient-centred oral healthcare. It would also be helpful to assess these differences in interventional studies for OLP following the core outcome set developed by the World Workshop on Oral Medicine VIII group: the appearance and severity of lesions, symptoms, function, social and psychological impacts, adverse effects, timelines, need for rescue medication and patient compliance, tolerability and satisfaction [32].

Concerning the oral premalignant and malignant changes, Thongprasom and colleagues (2010) noted that only 1.7% of 533 OLP records showed dysplasia. Another retrospective study also indicated a malignancy rate of 2.6% out of 384 patients with OLP/OLL [7]. Furthermore, the 10-year transformation rates of OLP and OLL were 1.2% and 4.4%, respectively [23]. Suggested risk factors included the site (tongue), the atrophic/ulcerative lesions, longer duration, tobacco use, alcohol drinking and hepatitis C infection [10,33]. Also, male gender and increased age were presently associated with dysplasia despite the lack of statistical significance of the latter. Following these findings, OLP lesions in males were indeed likely to have a more severe clinical form of OLP/OLL [23], showing dysplasia [34] and possibly progressing to OSCC [33]. Although, the MT is often seen in females—as noted among different longitudinal studies [23,35,36,37]. These differences are likely due to heterogeneous study methods (e.g., retrospective or prospective assessment), number of biopsies (e.g., frequent biopsy sampling with more severe diseases), follow-up period, exclusion criteria (e.g., excluding individuals with risk factors such as tobacco use and alcohol drinking) and geographical region [33,35,37]. 

Notably, around 25% of patients with OLP use tobacco and/or drink alcohol, which can act as independent risk factors for dysplastic changes and malignancy of the oral mucosa [4]. Unlike the inconclusive evidence on the malignancy potential of OLP lesions, the molecular investigations indicated a likely increased risk with dysplasia-associated OLM [11,15]. Whether the transformation to OSCC occurs instantly in these oral mucosal lesions or following the dysplastic changes remains in question [23]. 

The present study included a detailed analysis of histological findings and assessed their correlations with demographics and clinical variables of patients with OLP/OLL. Similar studies have primarily focused on demographics and clinical variables [16,38]. Also, the number of reviewed records was similar to global studies that reported 110–171 records [16,17,39]. 

Nevertheless, the present study was limited by its cross-sectional methods and lacked clinical correlation in 18% of the reviewed records, which does not necessarily exclude other oral disorders presenting lichenoid features other than OLP/OLL. Moreover, conducting separate analyses for OLP and OLL records was not possible due to the lack of clinical correlation and globally agreed diagnostic criteria for both conditions [9,12,40]. Important clinical variables such as medical history (e.g., list of medications), OLP clinical form (e.g., reticular, plaque-like, papular, bullous, atrophic and erosive), local risk factors (e.g., amalgam fillings), and involved extra-oral sites were frequently missing. Furthermore, the present study lacks the analysis of genetic polymorphism of inflammatory cytokines known for associations with susceptibility to OLP as performed in another Saudi patient cohort [41].

Therefore, future national and regional studies may consider further determining the clinicopathological correlations and assessing clinical [e.g., oral health indices, disease extent and severity scales, the rate and time from diagnosis to progression, number of affected sites and management outcomes], histological [e.g., percentage of dysplastic or malignant changes over time] and healthcare system-related variables [e.g., characteristics of referrals] [4,17,20,23,42]. 

Oral Medicine clinicians would also need to consider a multidisciplinary approach with dermatology and obstetrics-gynaecology specialists for the sometimes-extra-oral involvement of lichen planus as seen presently among 3 of the 140 patients [43]. Finally, national and perhaps regional patient registries for OLP and OLM could help to investigate aspects related to the patient (e.g., comorbidities, information and care needs and common concerns), their disease (e.g., number of affected sites, management outcomes and new drug discovery) and healthcare services (e.g., utilisation and quality of care). Furthermore, future studies may consider utilising machine and deep learning algorithms to analyse clinical images and histological slides to determine and predict the risk of MT in these oral mucosal lesions [44].

## 5. Conclusions

The present study findings demonstrated similarity to results from other retrospective studies that assessed patients’ demographics and clinicohistological characteristics with OLM, except for less likely reporting of oral symptoms. Buccal mucosa, gingiva and tongue were the most affected sites. Most oral biopsies showed band-like inflammatory cells, atrophy, basal cell degeneration and squamatisation. Despite their relatively low prevalence, the evidence of dysplastic changes in 12% of the 140 examined oral biopsy records may support its premalignant potential.

Thus, clinicians may consider addressing this limited risk with patients upon discussing histologically confirmed OLM. It would also be worthwhile to consider periodic reviews for early detection of changes in the oral mucosa, especially in patients with older age and active disease. Further prospective multi-centre studies with long follow-up periods would be needed to determine the associations between the demographic and clinicohistological characteristics of individuals with OLM and predict their risk of developing OSCC.

## Figures and Tables

**Table 1:** The clinical characteristics of screened patients’ records (n = 140).

Clinical Characteristics	Category	Number (%)
Associated symptoms	Asymptomatic	104 (74%)
	Burning sensation	15 (10%)
	Pain	19 (13%)
	Cutaneous pruritus	1 (0.7%)
	Nausea and vomiting	1 (0.7%)
Consistency	Soft	62 (44%)
	Firm	9 (6%)
	Not recorded	69 (49%)
Site	Buccal mucosa and sulcus	79 (56%)
	Ginigva and alveolar mucosa	21 (15%)
	Tongue	20 (14%)
	Lateral tongue	12 (8%)
	Dorsal tongue	4 (2%)
	Ventral tongue	2 (1%)
	Lips and labial mucosa	10 (7%)
	Hard and soft palate	7 (5%)
	The floor of the mouth	2 (1%)
Type of Biopsy	Incisional	135 (96%)
	Excisional	5 (3%)
Management	Corticosteroid alone or combined with immunosuppressants	22 (15%)
	Not required or recorded ^1^	117 (83%)

^1^ The management was unnecessary or recorded, as some patients were managed in other clinical settings.

**Table 2:** The histological findings of reviewed patients’ records (n = 140).

Findings	Number (%) of Records Showing this Finding	
	Yes	No
**Band-like inflammatory cells**	140 (100%)	0 (0%)
**Epithelial hyperkeratosis ^1^**	138 (99%)	2 (1%)
**Atrophic ^2^**	124 (88%)	15 (10%)
**Basal cell degeneration**	124 (88%)	16 (11%)
**Squamatisation**	122 (87%)	18 (12%)
**Thickening of basement membrane**	102 (72%)	38 (27%)
**Fibrin deposit ^3^**	100 (71%)	40 (28%)
**Civatte bodies**	85 (60%)	55 (39%)
**Melanin incontinence**	82 (58%)	58 (41%)
**Saw tooth rete ridges**	63 (45%)	77 (55%)
**Hypergranulosis**	57 (40%)	83 (59%)
**Artificial cleft formation**	53 (37%)	87 (62%)
**Acanthosis ^1^**	33 (23%)	106 (75%)
**Ulcer**	23 (16%)	117 (83%)
**Dysplastic changes**	18 (12%)	122 (87%)

^1^ Including hyperorthokeratosis, hyperparakeratosis, or both findings. ^2^ One record could not be assessed due to an artefact. ^3^ This finding was demonstrated using the direct immunofluorescence technique.

**Table 3:** Associations between medical history and presence of oral symptoms.

Medical History	Symptoms		*p*-Value
	Asymptomatic	Symptomatic	
Diabetes millets	15 (14.42%)	12 (33.33%)	0.0132 ^C^*
Hypertension	7 (6.73%)	13 (36.11%)	< 0.001 ^C^*
Hypothyroidism	6 (5.77%)	3 (8.33%)	0.6944 ^F^
Discoid lupus erythematous	6 (5.77%)	5 (13.89%)	0.1502 ^F^
Cutaneous lichen planus	3 (2.88%)	0	0.5692 ^F^
Asthma	2 (1.92%)	1 (2.78%)	0.9999 ^F^
Cutaneous allergy	2 (1.92%)	0	0.9999 ^F^
Breast cancer	0	2 (5.56%)	0.0648 ^F^
Cardiac catheterization	1 (0.96%)	0	0.9999 ^F^
Peptic ulcer	1 (0.96%)	0	0.9999 ^F^
Sjogren’s syndrome	1 (0.96%)	0	0.9999 ^F^
Psoriasis	1 (0.96%)	0	0.9999 ^F^
Liver cirrhosis	1 (0.96%)	0	0.9999 ^F^
Vitamin D deficiency	1 (0.96%)	0	0.9999 ^F^
Fatigue	1 (0.96%)	0	0.9999 ^F^
Depression	1 (0.96%)	1 (2.78%)	0.4495 ^F^
Inflammatory bowel disease	1 (0.96%)	0	0.9999 ^F^
Osteoporosis	1 (0.96%)	1 (2.78%)	0.4495 ^F^
Coronary artery bypass surgery	1 (0.96%)	0	0.9999 ^F^
Dry mouth	0	1 (2.78%)	0.2571 ^F^
Rheumatoid arthritis	0	1 (2.78%)	0.2571 ^F^

Abbreviations: ^C^—Chi-square test, ^F^—Fisher’s Exact test. * Indicates statistical significance.

**Table 4:** The significant associations between age and clinicohistological findings.

Variables		Age (Years)		*p*-Value
		Mean (±SD)	Median (Min, Max)	
Symptoms	Symptomatic	53.64 (±13)	56 (22, 79)	< 0.001 ^t^*
	Asymptomatic	45.24 (±12)	45 (22, 76)	
Ulcer	No	46.47 (±13)	47.5 (22, 79)	0.0149 ^t^*
	Yes	54.2 (±11)	52 (40, 75)	

Abbreviations: ^t^—Two sample *t*-test, * Indicates statistical significance.

## Data Availability

The datasets used and analysed during the current study are available from the corresponding author on reasonable request.

## Supplementary Materials

The following supporting information can be downloaded at: https://www.mdpi.com/article/10.3390/jcm12196383/s1, Supplementary File S1. The associations between gender and clinicopathological variables. Supplementary File S2. The non-statistically significant associations between age and other study variables.

## Abbreviations

MT	malignant transformation
OLL	oral lichenoid reaction
OLM	oral lichenoid mucositis
OLP	oral lichen planus
OSCC	oral squamous cell carcinoma
