# Supplementary material for: Clinicohistological Characteristics of Patients with Oral Lichenoid Mucositis: A Retrospective Study for Dental Hospital Records
Source: J Clin Med. 2023 Oct 6;12(19):6383. doi: 10.3390/jcm12196383 (PMC10573176; doi:10.3390/jcm12196383)
Supplement: Supplementary file 1 [file jcm-12-06383-s001.zip › jcm-2626638 - ori.docx]

Supplementary File 1. The associations between gender and clinicopathological variables.

| **Variables** | | **Gender** | | **p-value** |
| --- | --- | --- | --- | --- |
|  |  | **Female** | **Male** |  |
| Site | Alveolar mucosa | 0 | 1 (1.69%) | 0.4214^F^ |
|  | Alveolar ridge | 0 | 1 (1.69%) | 0.4214^F^ |
|  | Bilateral buccal mucosa | 2 (2.47%) | 0 | 0.5088^F^ |
|  | Bilateral tongue | 1 (1.23%) | 0 | 0.9999^F^ |
|  | Buccal mucosa | 46 (56.79%) | 28 (47.46%) | 0.2747^C^ |
|  | Buccal sulcus | 1 (1.23%) | 1 (1.69%) | 0.9999^F^ |
|  | Floor of Mouth | 1 (1.23%) | 1 (1.69%) | 0.9999^F^ |
|  | Gingiva | 12 (14.81%) | 4 (6.78%) | 0.1401^F^ |
|  | Hard palate | 2 (2.47%) | 4 (6.78%) | 0.2398^F^ |
|  | Labial mucosa | 5 (6.17%) | 4 (6.78%) | 0.9999^F^ |
|  | Lower right lip | 1 (1.23%) | 0 | 0.9999^F^ |
|  | Retromolar area | 1 (1.23%) | 2 (3.39%) | 0.5729^F^ |
|  | Right buccal mucosa | 0 | 1 (1.69%) | 0.4214^F^ |
|  | Soft palate | 0 | 1 (1.69%) | 0.4214^F^ |
|  | Tongue (dorsum) | 1 (1.23%) | 3 (5.08%) | 0.31^F^ |
|  | Tongue (lateral) | 7 (8.64%) | 5 (8.47%) | 0.9721^C^ |
|  | Tongue (non-Specific) | 1 (1.23%) | 0 | 0.9999^F^ |
|  | Tongue (ventral) | 0 | 2 (3.39%) | 0.1758^F^ |
| Symptoms | Asymptomatic | 57 (70.37%) | 47 (79.66%) | 0.2142^C^ |
|  | Symptomatic | 24 (29.63%) | 12 (20.34%) |  |
| Type of Biopsy | Excisional | 1 (1.23%) | 4 (6.78%) | 0.1619^F^ |
|  | Incisional | 80 (98.77%) | 55 (93.22%) |  |
| Squamatisation | No | 10 (12.35%) | 8 (13.56%) | 0.8322^C^ |
|  | Yes | 71 (87.65%) | 51 (86.44%) |  |
| Basal Cell Degeneration | No | 8 (9.88%) | 8 (13.56%) | 0.4989^C^ |
|  | Yes | 73 (90.12%) | 51 (86.44%) |  |
| Grade of inflammation | Mild | 15 (18.52%) | 12 (20.34%) | 0.1235^F^ |
|  | Moderate | 38 (46.91%) | 34 (57.63%) |  |
|  | High | 0 | 2 (3.39%) |  |
|  | Severe | 28 (34.57%) | 11 (18.64%) |  |
| Fibrin Deposit | No | 24 (29.63%) | 16 (27.12%) | 0.7454^C^ |
|  | Yes | 57 (70.37%) | 43 (72.88%) |  |
| Epithelial hyperkeratosis | No | 1 (1.23%) | 1 (1.69%) | 0.4381^F^ |
|  | Ortho | 9 (11.11%) | 8 (13.56%) |  |
|  | Ortho/para | 21 (25.93%) | 20 (33.9%) |  |
|  | Para | 50 (61.73%) | 30 (50.85%) |  |
| Hyper granulosis | No | 52 (64.2%) | 31 (52.54%) | 0.1657^C^ |
|  | Yes | 29 (35.8%) | 28 (47.46%) |  |
| Acanthosis | No | 66 (81.48%) | 40 (68.97%) | 0.0873^C^ |
|  | Yes | 15 (18.52%) | 18 (31.03%) |  |
| Atrophic | No | 8 (9.88%) | 7 (12.07%) | 0.6812^C^ |
|  | Yes | 73 (90.12%) | 51 (87.93%) |  |
| Civatte Bodies | No | 29 (35.8%) | 26 (44.07%) | 0.3228^C^ |
|  | Yes | 52 (64.2%) | 33 (55.93%) |  |
| Saw Tooth Rete Ridges | No | 45 (55.56%) | 32 (54.24%) | 0.8770^C^ |
|  | Yes | 36 (44.44%) | 27 (45.76%) |  |
| Artificial Cleft Formation | No | 49 (60.49%) | 38 (64.41%) | 0.6374^C^ |
|  | Yes | 32 (39.51%) | 21 (35.59%) |  |
| Melanin Incontinence | No | 31 (38.27%) | 27 (45.76%) | 0.3743^C^ |
|  | Yes | 50 (61.73%) | 32 (54.24%) |  |
| Dysplastic Changes | No | 75 (92.59%) | 47 (79.66%) | 0.024^C^* |
|  | Yes | 6 (7.41%) | 12 (20.34%) |  |
| Low/High OED Grading | Mild | 6 (7.41%) | 11 (18.64%) | 0.0473^F^* |
|  | No | 75 (92.59%) | 47 (79.66%) |  |
|  | Verrucous hyperplasia/cytological atypia | 0 | 1 (1.69%) |  |
| Thickening of Basement Membrane | No | 23 (28.4%) | 15 (25.42%) | 0.6963^C^ |
|  | Yes | 58 (71.6%) | 44 (74.58%) |  |
| Ulcer | No | 68 (83.95%) | 49 (84.48%) | 0.9325^C^ |
|  | Yes | 13 (16.05%) | 9 (15.52%) |  |

Abbreviations: C – Chi square test, F – Fisher’s Exact test,. ** indicates statistical significance.*

Supplementary File 2. The non-statistically significant associations between age and other study variables.

| **Variables** | | **Age (years)** | | **p-value** |
| --- | --- | --- | --- | --- |
|  |  | **Mean ± SD** | **Median (Min, Max)** |  |
| Squamatisation | No | 47.5 ± 11.76 | 49 (30, 74) | 0.9969^t^ |
|  | Yes | 47.51 ± 13.44 | 48 (22, 79) |  |
| Basal cell degeneration | No | 45.5 ± 9.53 | 49 (30, 61) | 0.4044^t^ |
|  | Yes | 47.79 ± 13.61 | 48 (22, 79) |  |
| Fibrin deposit | No | 48.44 ± 11.22 | 50 (23, 69) | 0.4749^MW^ |
|  | Yes | 47.16 ± 13.88 | 46 (22, 79) |  |
| Hyper granulosis | No | 47.47 ± 13.31 | 48 (22, 75) | 0.9644^t^ |
|  | Yes | 47.57 ± 13.12 | 49 (24, 79) |  |
| Acanthosis | No | 48.47 ± 12.84 | 49 (22, 79) | 0.2037^t^ |
|  | Yes | 45.03 ± 13.89 | 45 (22, 76) |  |
| Atrophic | No | 46 ± 13.31 | 49 (24, 65) | 0.6048^t^ |
|  | Yes | 47.87 ± 13.14 | 48 (22, 79) |  |
| Civatte bodies | No | 46.29 ± 11.31 | 45 (28, 74) | 0.3696^t^ |
|  | Yes | 48.3 ± 14.27 | 50 (22, 79) |  |
| Saw tooth rete ridges | No | 47.49 ± 11.73 | 47.5 (26, 79) | 0.8142^MW^ |
|  | Yes | 47.54 ± 14.81 | 49 (22, 76) |  |
| Artificial cleft formation | No | 45.99 ± 12.24 | 45 (22, 79) | 0.0706^MW^ |
|  | Yes | 49.96 ± 14.36 | 53 (22, 76) |  |
| Melanin incontinence | No | 49.58 ± 12.79 | 50 (22, 75) | 0.1287^t^ |
|  | Yes | 46.05 ± 13.34 | 45.5 (22, 79) |  |
| Dysplastic changes | No | 47.27 ± 13.42 | 48 (22, 79) | 0.5949^t^ |
|  | Yes | 49.06 ± 11.79 | 49 (30, 70) |  |
| Thickening of Basement Membrane | No | 47.79 ± 12.47 | 50 (23, 76) | 0.8143^t^ |
|  | Yes | 47.41 ± 13.48 | 48 (22, 79) |  |

Abbreviations: t – Two sample t-test, MW – Mann Whitney U test. * Indicates statistical significance.
